# Supplementary material for: Implementation of a surgical ward innovation: Telemonitoring controlled by healthdot [SWITCH-trial PROTOCOL]
Source: PLoS One. 2025 May 7;20(5):e0322472. doi: 10.1371/journal.pone.0322472 (PMC12057926; doi:10.1371/journal.pone.0322472)

**SWITCH - Surgical Ward Innovation: Telemonitored controlled by Healthdot**

**Evaluation of implementation**

**SWITCH-project – Surgical Ward Innovation: Telemonitored Controlled by Healthdot**

**Subtitle: The evaluation of the implementation**

| **Short title** | **Healthdot on surgical ward** |
| --- | --- |
| **Version** | **2.0** |
| **Date** | **27-05-2025** |
| **Principal investigator(s)** | **Drs. F.M.J.F. Schonck (friso.schonck@catharinaziekenhuis.nl)** |
| **Investigator(s)** | **Dr. R.A. Bouwman (**[**Arthur.bouwman@catharinaziekenhuis.nl**](mailto:Arthur.bouwman@catharinaziekenhuis.nl)**)**  **Dr. S.W. Nienhuijs (**[**simon.nienhuijs@catharinaziekenhuis.nl**](mailto:simon.nienhuijs@catharinaziekenhuis.nl)**)**  **Dr. M.D.P. Luyer (**[**misha.luyer@catharinaziekenhuis.nl**](mailto:misha.luyer@catharinaziekenhuis.nl)**)**  **Prof. Dr. N.J.M van der Meer (**[**nardo.vd.meer@catharinaziekenhuis.nl**](mailto:nardo.vd.meer@catharinaziekenhuis.nl)**)** |
| **Investigational site** | **Department of Surgery, Catharina Hospital, Michelangelolaan 2, 5623 EJ Eindhoven, The Netherlands** |
| **Other participating sites** | **N/A** |
|  |  |
| **Sponsor** | **Catharina hospital Eindhoven** |
|  |  |
| **Independent expert (s)** | Evert Koldewijn, Urologist at Urology Department, Catharina Hospital, Eindhoven |
| **Laboratory sites** | **N/A** |

**PROTOCOL SIGNATURE SHEET**

| **Name** | **Signature** | **Signature + Date** |
| --- | --- | --- |
| **Head of Department** | **Nardo van der Meer** |  |
| **Principal investigator** | **Friso Schonck** |  |
| **Investigator** | **Simon Nienhuijs** |  |
| **Investigator** | **Arthur Bouwman** |  |
| **Investigator** | **Misha Luyer** |  |
| **Investigator** | **Nardo van der Meer** |  |
| **Additional/other** | | |
| **Author** |  |  |
| **Reviewer** |  |  |
| **Reviewer** |  |  |

**TABLE OF CONTENTS**

APPROVAL/ PROTOCOL SIGNATURE SHEET 2

TABLE OF CONTENTS 4

LIST OF ABBREVIATIONS AND RELEVANT DEFINITIONS 5

SUMMARY 6

1. INTRODUCTION AND RATIONALE 10

2. OBJECTIVES 12

3. STUDY DESIGN 14

4. PROJECT POPULATION 14 4.1 Population (base) 15 4.2 Inclusion criteria 16 4.3 Exclusion criteria 16 4.4 Sample size calculation 16

5. TREATMENT OF SUBJECTS 16

6 Investigational product/treatment 16

6.1 Name and description of non-investigational product(s) 16

6.2 Summary of findings from non-clinical studies 18

6.3 Summary of known and potential risks and benefits 18

7. METHODS 187.1 Study parameters/endpoints 18 7.1.1 Main study parameter/endpoint 18 7.1.2 Secondary study parameters/endpoints (if applicable) 19 7.1.3 Other study parameters (if applicable) 19

7.2 Randomisation, blinding and treatment allocation 20

7.3 Study procedures 21

7.4 Withdrawal of individual subjects 22

7.5 Replacement of individual subjects after withdrawal 22

7.6 Follow-up of subjects withdrawn from treatment 22

7.7 Premature termination of the study 22

8 STATISTICAL ANALYSIS 23 8.1 Other study parameters 23

9. ADMINISTRATIVE ASPECTS, MONITORING AND PUBLICATION 23

9.1 Handling and storage of data and documents 23 9.2 Monitoring and Quality Assurance 24 9.3 Amendments 24 9.4 Annual progress report 24 9.5 End of study report 24 9.6 Public disclosure and publication policy 24

10. REFERENCES 25

11. ATTACHMENTS 27

**LIST OF ABBREVIATIONS AND RELEVANT DEFINITIONS**

| **ABR** | **General Assessment and Registration form (ABR form), the application form that is required for submission to the accredited Ethics Committee; in Dutch: Algemeen Beoordelings- en Registratieformulier (ABR-formulier)** |
| --- | --- |
| **AE** | **Adverse Event** |
| **AR** | **Adverse Reaction** |
| **CA** | **Competent Authority** |
| **CCMO** | **Central Committee on Research Involving Human Subjects; in Dutch: Centrale Commissie Mensgebonden Onderzoek** |
| **CV** | **Curriculum Vitae** |
| **DSMB** | **Data Safety Monitoring Board** |
| **EWS** | **Early warning score** |
| **GCP** | **Good Clinical Practice** |
| **GDPR** | **General Data Protection Regulation; in Dutch: Algemene Verordening Gegevensbescherming (AVG)** |
| **IB** | **Investigator’s Brochure** |
| **IC** | **Informed Consent** |
| **Investigational site** | **Institution or site where the clinical investigation is carried out** |
| **IRB** | **Institutional review board** |
| **METC** | **Medical research ethics committee (MREC); in Dutch: medisch-ethische toetsingscommissie (METC)** |
| **Sponsor** | **The sponsor is the party that commissions the organisation or performance of the research, for example a pharmaceutical**  **company, academic hospital, scientific organisation or investigator. A party that provides funding for a study but does not commission it is not regarded as the sponsor, but referred to as a subsidising party.** |
| **WMO** | **Medical Research Involving Human Subjects Act; in Dutch: Wet Medisch-wetenschappelijk Onderzoek met Mensen** |

**SUMMARY**

**Brief summary**:

Healthcare has undergone major development in recent decades due to the application of new technology. One of these developments concerns the monitoring of in-hospital admitted patients and remote monitoring of discharged patients with smart autonomous sensors (1). This technological development is highly desirable as the current manually performed monitoring is sub-optimal and has not undergone major development, unlike prevention, diagnosis and treatment, since the 90’s (2). Hospital care is currently in a transitional phase, which has been accelerated by the COVID-19 pandemic, to more outpatient care, shortened length of admissions and single day admission. The demographics of the hospitalized patients have therefore changed to a more vulnerable and comorbid population which makes them more prone for complications (3). On addition, the health care sector experiences staff shortages, especially within nursing care while the demand for hospital care expands. As a result, the workload is high, time is scarce and quality of care can be negatively influenced (4). Monitoring is according to current hospital protocol manually measured, three times a day and displayed as EWS score. This method is time-consuming, inefficient, subjective and error prone (3). Smart autonomous sensors have the potential, illustrated in previous research and literature, to cope with these challenges (5). However, implementation and integration of a new system is complex and must be carried out step by step with all those involved (6).

This study aims to step-by-step implement and evaluate an already validated smart monitoring device, the Philips Healthdot, on a hospital ward at the Catharina hospital.

**Rationale:** Ward monitoring is crucial in early recognition of complications and clinical deterioration. Smart autonomous monitoring enables continuous observation, is expected to reduce nursing workload, improve efficiency without compromise in confidence and might cause earlier recognition of clinical decline.

**Objective**:

Primary objectives

Evaluation of the implementation of a continuous monitoring method on the surgical ward

- The degree to which the Healthdot monitoring method is implemented as originally planned
- Average amount of manual spot checks per hospital admission day

Secondary objectives

- The intention and attitude towards the use of the Healthdot monitoring method.
- The extent to which the Healthdot monitoring method can be successfully carried out by the healthcare employees
- Amount of manual spot checks per hospital admission day per patient
- The perception among nursing staff given the satisfactory and agreeableness regarding workload.
- Relation between device false alarms (noise) and correct alarms based on the alarming protocol.
- Number of unexpected events that occur during or result from the use of the Healthdot medical device.
- The amount of time needed to perform and process a complete manual spot check or digital monitoring check.
- Cost-effectiveness analysis based on the duration of admission, the associated admission costs and the monitoring method
- Advanced algorithm analyses for improved monitoring protocols

**Project design:**

This project is a 6 months monocenter prospective cohort at the Catharina hospital in the Netherlands. Every patient admitted to the one surgery department will receive a Healthdot. Implementation of the Healthdot will be performed stepwise. During phase 1 (month 1-3, the pre-implementation phase), the current standard method of care with manual spot checks and EWS will be continued. During this phase Healthdots will be applied only for training purposes in preparation to the implementation phase, phase 2 (month 3-6). This phase consists of primarily Healthdot monitoring and manual spot checks only if indicated. After these 6 months, the implementation will be evaluated and optimized and potentially extended.

**Participants:**

The main research population for answering the research objectives are the nurses and physicians working at the specific surgical ward at the Catharina hospital.

In total around 500 patients, 250 during each phase, admitted to one surgical ward at the Catharina hospital will participate and receive a Healthdot as the new standard of care. These patients will sign informed consent for use of anonymized data. This population differs in age, gender, admission cause, admission duration and comorbidities but reflects the demographics of the general population on this ward which is essential to investigate implementation and prevent selection bias.

**Main implementation study parameters/endpoints:**

The medical research council (MRC) provided a document in 2000, the MRC model, to help researchers use the correct research designs, methods and measuring instruments (6). This model was revised in 2021 and forms the basis of the design of this project (6). This model structures the analysis using psychometric properties such as: acceptability, adoption, appropriateness, costs, feasibility, fidelity, penetration and sustainability (7).

Primary endpoints

Evaluation of the implementation of a continuous monitoring method on the surgical ward

- Fidelity: The usability and degree of implementation of the Healthdot at the surgical ward. Measured with the System usability scale (SUS), Score > 68 at M6 (8)
- Acceptability: The workload regarding the amount of spot checks in addition to the standard monitoring. Calculated with data from the electronic patient file records. Success in case of average reduction of 20% at M6 compared to M3

Secondary endpoints

- Adoption: The intention and attitude towards the use of the Healthdot monitoring method. Measured with evidence-based practise attitude scale (EBPAS)(9, 10).
- Feasibility: The extent to which the Healthdot monitoring method can be successfully carried out by the healthcare employees. Measured with a thematic analysis and input from the core group (Braun and Clark)(11)
- Acceptability: The workload regarding the amount of spot checks per hospital admission day per patient. Calculated with data from the electronic patient file records.
- Acceptability: The perception among nursing staff given the satisfactory and agreeableness regarding workload. Measured with the integrated workload scale (IWS) (12, 13).
- Appropriateness: Relation between device false alarms (noise) and correct alarms based on the alarming protocol. Measured as signal to noise ratio (SNR)
- Appropriateness: Number of unexpected events that occur during or result from the use of the Healthdot medical device. Measured as adverse device events (ADE)
- Feasibility: The amount of time needed to perform and process a complete manual spot check or digital monitoring check. Measured by manual time measurement.
- Costs: Cost-effectiveness analysis based on the duration of admission, the associated admission costs and the monitoring method. Calculation based on provided care and associated costs
- Algorithm improvement: Advanced algorithm analyses for improved monitoring protocols with use of artificial intelligence (AI)

**Nature and extent of the burden and risks associated with participation, benefit and group relatedness:**

This implementation study is a quality project in collaboration with the quality and care department at the Catharina hospital as part of to the transition of care.

Nursing staff and physicians will be trained to work with the Healthdot and its dashboard to evaluate the readings. The implementation of a new system, especially in healthcare, is challenging because of sometimes interfering interests and great responsibilities (6). With use of the stepwise approach and continuous evaluation with all involved, benefits such as reduction in workload and qualitative vial parameter live readings will be apparent (14).

Admitted patients receiving the Healthdot might, in rare cases, experience allergic symptoms or discomfort wearing the Healthdot although these side effects are very rare in previous research (15-17). Potential chemical, electrical and biological hazards have not been identified(15-17). Patient related benefits include improved confidence throughout continuous monitoring, less impact on day-night rhythm compared to manual spot checks and more time for non-medical patient care (18).

#

# 1. INTRODUCTION AND RATIONALE’

the healthcare sector is a complex system which is currently in a transitional phase. As part of this transition there are changes and developments in the field of new systems and techniques. Implementation of such a new system within the healthcare system is challenging and still very limited investigated (19). Implementation of a new system within a multidisciplinary team is a enormous challenge and must be carried out step-by-step. For this reason, the medical research council (MRC) provided a document in 2000, the MRC model, to help researchers use the correct research designs, methods and measuring instruments (6). This model was revised in 2021 and forms the basis of the design of this project, aimed at implementing a smart monitoring sensor at the surgical department (6).

Medical devices to monitor vital parameters were introduced during the 20^th^ century. Around 1990 routine monitoring with use of a universal scoring system, early warning score (EWS) was proposed. EWS was introduced as a potential solution for the high incidence of adverse events and preventable deaths in hospitalized patients (2, 3). This system was designed to identify the likelihood of patients deteriorating during hospitalization (2). Clinical decline is known to be preceded by changes in vital parameters. Early recognition of these alterations is crucial because early start of the appropriate treatment enhances the outcome of the patient (20). Postoperative complications are frequently encountered at the surgical ward. These complications are associated with a longer length of stay and can lead to major adverse events such as unplanned intensive care unit (ICU) admissions, cardiorespiratory arrest or even death (21). 60% of the patients with these major complications showed prior to the event vital sign deterioration which is frequently not recognized by medical staff or spot check monitoring (2).

Over the years various improvements have been made to better stratify the risk for complications in the pre-and intra-operative setting. Vital signs are collected pre-operative and continuously during surgery. Postoperative monitoring at the hospital ward consist of three spot checks a day and consists of heart rate, respiratory rate, oxygen saturation, blood pressure, temperature and pain scores. These parameters are scored and displayed as an overall EWS score. Technological developments introduced possibilities to electrify some of these vital parameter measurements with enhanced accuracy as a result (5, 15, 22). The original EWS system was, based on new insights, improved and replaced for the modified EWS (MEWS) which included oxygen saturation and the national EWS (NEWS) which included the use of supplemental oxygen saturation (20, 21). Despite these advances, EWS methodological outcomes are moderate and discrimination is poor in daily practise (2, 3). Additionally, the application of many different subtypes is widespread and validation studies contain methodological and reporting shortcomings (2, 3). These factors question the clinical reliability of EWS and require a development regarding monitoring methods.

In the Catharina hospital, two studies were conducted with the Philips Healthdot, the PEACH (16) study and the TRICA study (15, 17). The TRICA study, performed at the surgical department, contributed to the CE-marking of the Healthdot as a monitoring device. The PEACH study has investigated outpatient monitoring after bariatric surgery with a positive result(15, 17).

With the transition to surgery in ambulatory setting, accelerated by the COVID-19 pandemic, the hospitalized patients are older, have more comorbidities and are therefore more prone to complications. Early vital sign alterations such as tachypnoea and tachycardia are common underdiagnosed in post-operative surgery patients (22). Automated monitoring is able to identify these micro-events and thereby offers the potential to improve ward monitoring and the medical care (22).

The application of smart monitoring devices as a solution for these limitations is a hot topic within the transition of care. Many domestic and international study groups have demonstrated the effectiveness of a smart monitoring sensor in improved patient monitoring on the hospital ward (5, 14, 23-25).

Concluding, automated monitoring has great potential for improvement of hospital ward care. Implementation of such a new monitoring system is challenging and requires a careful stepwise approach. This study will investigate whether the implementation of the Healthdot can be achieved in a 6-month period.

# OBJECTIVES

**Objective**:

Primary objectives

Evaluation of the implementation of a continuous monitoring method on the surgical ward

- Fidelity: The usability and degree of implementation of the Healthdot at the surgical ward
- Acceptability: The workload regarding the amount of spot checks in addition to the standard monitoring.

Secondary objectives

- Adoption: The intention and attitude towards the use of the Healthdot monitoring method.
- Feasibility: The extent to which the Healthdot monitoring method can be successfully carried out by the healthcare employees
- Acceptability: The workload regarding the amount of spot checks per hospital admission day per patient
- Acceptability: The perception among nursing staff given the satisfactory and agreeableness regarding workload.
- Appropriateness: Relation between device false alarms (noise) and correct alarms based on the alarming protocol.
- Appropriateness: Number of unexpected events that occur during or result from the use of the Healthdot medical device.
- Feasibility: The amount of time needed to perform and process a complete manual spot check or digital monitoring check.
- Costs: Cost-effectiveness analysis based on the duration of admission, the associated admission costs and the monitoring method
- Advanced algorithm analyses for improved monitoring protocols

Primary hypothesis:

Previous own research (TRICA-trial, PEACH-trial) (15, 17) has shown that the Healthdot monitoring device is non-inferior to manual monitoring with EWS on the surgical ward regarding predicting and alarming of deteriorating patients based on vital parameter alterations. Our hypothesis is that implementation of this new monitoring method is a challenge but that a stepwise and careful approach, with involvement of all healthcare staff, will have a positive effect on the nursing workload and efficiency without reduction in confidence and thereby improvement of the overall workability and efficiency at the surgical ward.

Secondary hypothesis:

We hypothesize that the implementation of het Healthdot will be accepted and supported by the nursing staff and physicians since previous research has generated a positive attitude towards the use of the Healthdot in our hospital. Our assumption is that the attitude towards this change will shift towards an even more positive mindset during the implementation phase and that the training prior to this will have an important contribution. This change will be measured by the questionnaires, calculations and the Braun and Clarke thematic analysis. The device performance and signal-to-noise ratio and adverse device events will be analysed and are not expected to adversely affect the workability based on previous research.

These hypotheses will be accepted or rejected based on the outcome of this clinical investigation.

# Project DESIGN

This project is designed as a 6-month monocentric prospective cohort at the Catharina hospital in the Netherlands. In the presently unlikely event that other sites will be included during the conduct of het study, amendments will be submitted to all respective internal and external approvers (ICBE, METC). Every patient admitted to the one surgery department during the duration of the study will receive a Healthdot as monitoring device. Implementation of the Healthdot as the new standard of care regarding monitoring will be performed stepwise in different phases: pre-condition phase, pre-implementation phase, implementation phase and maintenance and evolution.

During phase 1 (month 1-3) patients will receive the current standard monitoring method with manual spot checks and EWS. During this phase all healthcare staff involved will receive appropriate training regarding application of the device, reading and assessing the data and protocol explanation to optimize the infrastructure before implementation.

During phase 2 (month 4-6) patients will be monitored with the Healthdot primarily and receive additional manual spot checks if indicated. After these 6 months, the implementation will be evaluated and optimized and potentially extended.


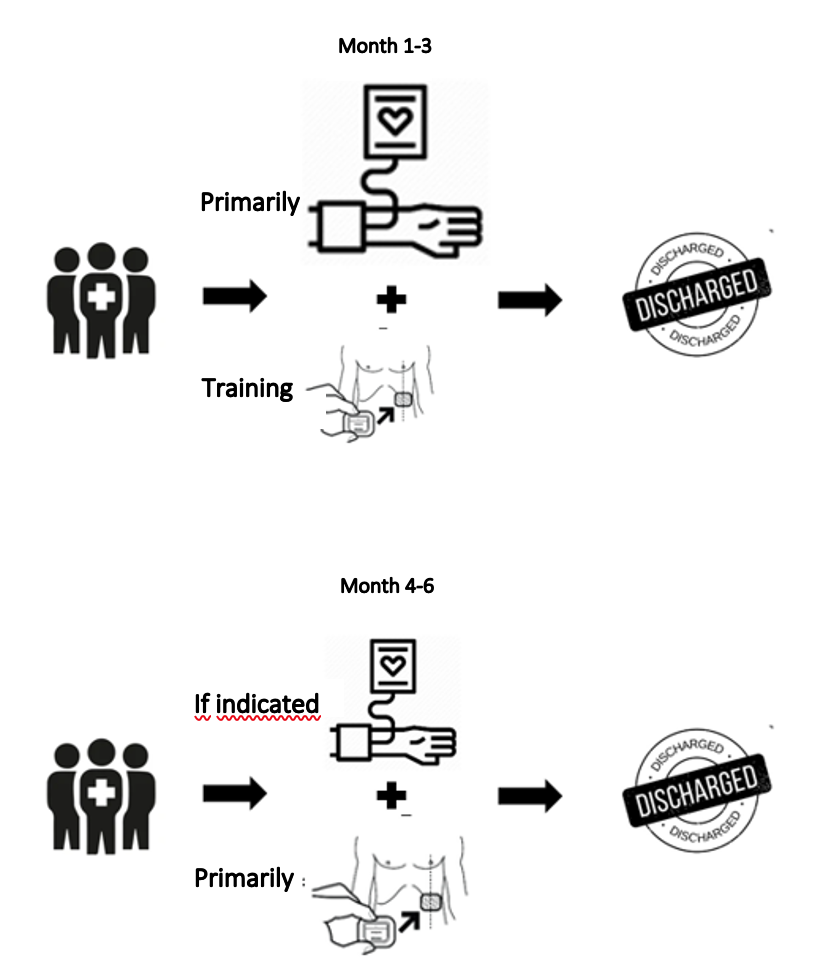


Figure 1: Flowchart project design

| Surgical ward | Month 1 | Month 2 | Month 3 | Month 4 | Month 5 | Month 6 |
| --- | --- | --- | --- | --- | --- | --- |
|  | Spot check + EWS | Spot check + EWS | Spot check + EWS | Healthdot | Healthdot | Healthdot |
| Focus | Training | Training | Training | Implementation | Implementation | Implementation |
|  | Infrastructure | Infrastructure | Infrastructure | Evaluation | Evaluation | Evaluation |
|  | Baseline data | Baseline data | Baseline data | Study data | Study data | Study data |

#
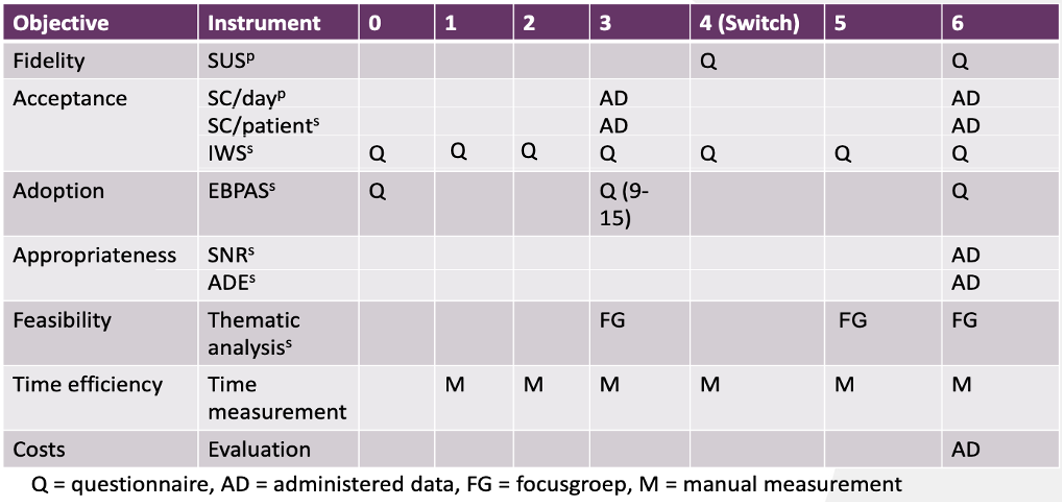
Table 1: Overview focus during project phases

FG

# Table 2: Measurement timetable for psychometric properties

# PROJECT POPULATION

## Population (base)

Around 10 nurses and 5 physicians, working at the designated surgical ward, will sign informed consent and participate as the primary research population in the core group for in depth analysis regarding the implementation evaluation.

Adults, male or female above 18 years old, admitted to one surgical ward at the Catharina hospital will be participating in this prospective cohort study. Admission will mainly concern postoperative patients, but readmissions, clinical observation, treatment of complications or transfers from other departments or the intensive care unit are also reasons for admission. Most of the patients that will be treated at this department receive care of oncological surgical nature, but also general surgical patients, urological and gynaecological patients can be admitted to this department.

This ward has an average annual turnover of approximately 900-1000 patients each year based on the previous three years. A study period of 6 months is proposed to obtain a total of 500 patients in total, 250 patients during each study phase.

## Inclusion criteria

Nurses and physicians are the primary research population for answering the research objectives.

Nurses and physicians, participating in het focus group will sign informed consent.

Nurses working at the surgical ward who will fill in the IWS questionnaire at multiple occasions will sign for informed consent on this questionnaire form (attachment 3) for use of the anonymized data.

Patients won’t be included for participation since the Healthdot will be the new standard of care regarding monitoring. Patients will however sign informed consent at admission for use of anonymized data.

Core/focus group

Nurses

- Registered in the national register of medical professions (Big register)
- Appointed as a nurse at the Catharina hospital and working in the surgical department during the study period.
- Willingness to participate in the core group for evaluation of the implementation

Physicians

- Registered in the national register of medical professions (Big register)
- Appointed at the Catharina hospital as a physician or resident.
- Willingness to participate in the core group for evaluation of the implementation

Nursing team working at this surgical ward will fill in the IWS on a weekly base. Every nurse will consent to the voluntary participation for this one question inquiry and consent to use of the anonymous data. No additional informed consent will be asked to this group.

## Exclusion criteria

Core/focus group

Nurses & Physicians

- Termination of employment within the study period
- Not willingness to participate in the core group

## Sample size calculation

Sample size calculation is not applicable for implementation projects. Nevertheless, we expect to obtain data from at least 500 patients, 250 patients during each phase, based on the average annual turnover of approximately 900-1000 patients each year over the previous three years at this ward. Our core group contains out of 10 nurses and 5 physicians.

# TREATMENT OF SUBJECTS

This study does not compare treatments the implementation of a new medical monitoring device which will be discussed in chapter 5.

#

# INVESTIGATIONAL PRODUCT

## Name and description of investigational product(s)

The Healthdot is a CE marked wearable data logger (15, 17). It consists of an

adhesive layer, electronics and a battery. The intern mechanism contains an accelerometer which is able to measure movement including movement of the lung and heart. This movement data is processed with a validated algorithm to calculate heart rate,

respiratory rate, posture and physical activity which are stored on the device and transferred

through the LoRa network via the local gateway and/or gateways

provided by KPN. The entire technological infrastructure system consists of:

1. Healthdot

2. Gateway

3. KPN-LoRa network

4. Server

5. Guardian dashboard


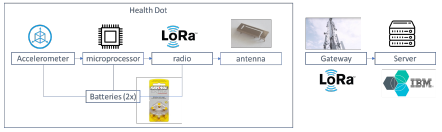


Required training

The investigators, nursing staff and physicians will receive a training and e-learning during the pre-implementation phase about the application of the Healthdot, the activation sequence and data reading. In addition, the structure of the study with the questionnaires, the timetable, the procedures in cases of malfunctions/ ADEs will be provided in a protocol.

## Contra-indications

- Known to have severe allergy for the tissue adhesive used in the Healthdot.
- Any skin condition, for example prior severe rash, discoloration, scars or open wounds at the area of application
- Pregnant, or breastfeeding
- Use of topical that is known to influence the skin at the test area (such as medical and nonmedical creams or lotions)
- Patient with active implantables such as Implantable Cardioverter Defibrillator (ICD) and pacemaker
- Unable to understand instructions
- Left lower rib (place where Healthdot will be applied) is involved in the area of surgery, area of disinfection or area where bandages are needed.
- Transfer to a different ward during hospitalization

Indications for switching Healthdot

- No successful measurement after 4 hours or earlier if clinically necessary.
- When the Healthdot is not connected within 60 min after application

Indications to remove the Healthdot

- In case that an exclusion criterion is met during the study

## Summary of findings from non-clinical studies

## Research into the implementation of a new technology in healthcare can only be investigated in a clinical setting. No non-clinical studies are therefore contributing.

## Summary of known and potential risks and benefits

Previous research and findings from the literature show that the use of the Healthdot as a monitoring device is a validated measurement method that is at least as effective as the manually performed measurements with EWS according to the protocols in the Catharina hospital.

Serious adverse device events have not been established and mild adverse device events such as hypersensitivity reactions or rash have been observed only to a limited extent (15). Application of the Healthdot at the surgical ward is therefore possible in a safe manner, with the aim to implement this new monitoring method. Clinical observation and any additional spot checks remain available if indicated and the stepwise structure ensures optimization of the infrastructure before the implementation starts. This procedure optimally guarantees security and implementation can be carried out within a secure framework.

# METHODS

## Study parameters/endpoints

### Main study parameter/endpoint

Primary parameters and endpoints

Evaluation of the implementation of a continuous monitoring method on the surgical ward

- Fidelity: The usability and degree of implementation of the Healthdot at the surgical ward
  - Measured with the System usability scale (SUS), Score > 68 at M6 (8, 26)
- Acceptability: The workload regarding the amount of spot checks in addition to the standard monitoring.
  - Calculated with data from the electronic patient file records. Success in case of average reduction of 20% at M6 compared to M3

### Secondary study parameters/endpoints (if applicable)

Secondary parameter and endpoints

- Adoption: The intention and attitude towards the use of the Healthdot monitoring method.
  - Measured with evidence-based practise attitude scale (EBPAS) (9, 10).
- Feasibility: The extent to which the Healthdot monitoring method can be successfully carried out by the healthcare employees.
  - Measured with a thematic analysis (Braun and Clark) with core group (11).
- Acceptability: Amount of manual spot checks per hospital admission day per patient
  - Calculated with data from the electronic patient file records.
- Acceptability: The perception among nursing staff given the satisfactory and agreeableness regarding workload.
  - Measured with the integrated workload scale (IWS) (12, 13).
- Appropriateness: Relation between device false alarms (noise) and correct alarms based on the alarming protocol.
  - Measured as signal to noise ratio (SNR)
- Appropriateness: Number of unexpected events that occur during or result from the use of the Healthdot medical device. Measured as adverse device events (ADE)
- Feasibility: The amount of time needed to perform and process a complete manual spot check or digital monitoring check. Measured manually.
- Costs: Cost-effectiveness analysis based on the duration of admission, the associated admission costs and the monitoring method
- Advanced algorithm analyses for improved monitoring protocols with advanced AI software

### Other study parameters (if applicable)

Additional anonymized data will, in case of informed consent, be collected from the electronic patient file such age, gender, reason for admission, medical history, duration of admission, complications and laboratory results. This additional data will be used to relate the primary and secondary results to any influencing factors/ baseline characteristics and potentially correct for these factors.

Variables eCRF

| Characteristic | Input |
| --- | --- |
| Indication | Open |
| Type of invasive interventions/surgery | Open |
| Gender | M/F |
| Age | Open |
| BMI | Kg/m2 |
| Comorbidities |  |
| Charlson comorbidity index | [Charlson Comorbidity Index (CCI) - MDCalc](https://www.mdcalc.com/charlson-comorbidity-index-cci) |
| Hospital stay |  |
| Admission date | D/M/Y |
| Discharge date | D/M/Y |
| Elective or emergency | Open |
| Complications |  |
| Clavien-Dindo classification | Grade I-V |
| Complications type | Cardiac, Pulmonary, Kidney failure, infectious, traumatic, surgical related (perforation, leakage, bleeding) |
| Urgent consultations (SIT) during hospitalization | 0-99 |
| ICU admission | Yes/no |
| Mortality <30 days | Yes/no |
| Re-admission < 30 days | Yes/no |
| Fidelity |  |
| Total minutes monitored | Min |
| Amount of heart rate measurements | Total number per admission |
| Amount of respiratory rate measurements | Total number per admission |
| Artefact heart rate measurements | Total number per admission |
| Artefact respiratory rate measurements | Total number per admission |
| Total amount of spot checks | Total number per admission |
| Device |  |
| Premature removal of device | Yes/no |
| Replacement of device | Yes/no |
| Bijzonderheden device | Yes/no |

## Randomisation, blinding and treatment allocation

N/A

## Project procedures

Prior to the start of the pre-implementation phase there will be a pre-implementation phase during months 1-2. This phase will consist of training the medical staff through e-learning and physical meetings. During this phase, medical staff will have the opportunity to apply the Healthdot, learn to activate it and practise with the Guardian dashboard. This phase serves as preparation for the implementation and aims to optimize the intra-structure in advance. In addition, during this phase, baseline data will be collected about the standard spot check measurements, feeling of confidence and attitude towards a change in the monitoring method. This study is a quality project and a change in standard of care. Nurses and physicians voluntarily participating in the focus group will sign informed consent for participation. Nurses not participating in the focus group but working at the surgical ward during this project will

During the first phase, the daily output will consist of:

- 3x daily spot check according to the hospital protocol where the vital values are manually entered in the electronic patient file and scored with EWS. In addition, the Healthdot will be applied for training purposes. Data from the Healthdot during this phase will not be used in clinical care.


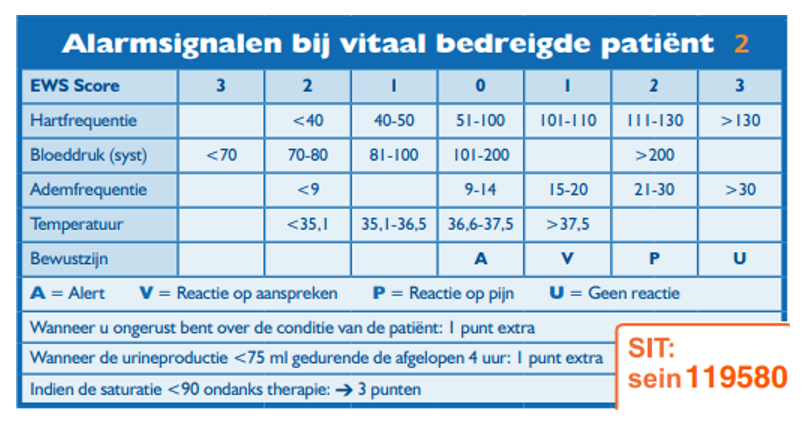
Table 2: Current (M)EWS protocol at the Catharina hospital for ward monitoring

- During phase 2, the Healthdot is the primary monitoring method. Each patient admitted to the surgical ward will receive the Healthdot during intake until discharge. In case the hospitalization duration is longer than the battery life, approximately 14 days, the Healthdot will be replaced by a new device. The Healthdot measures heart rate, respiration rate and physical activity every 5 minutes. These parameters are displayed in the Guardian dashboard as a trendline and automatically transferred to the electronic patient file 3 times a day (08u,12u,17u). These measurements are scored in accordance with the alarm protocol and, if necessary, provide a notification. In case of an additional measurement, a spot check will be performed by the nursing staff.

The protocol alarms in case the respiration rate is above 20/min and or the heart rate is 110 or more for 15 minutes consecutive.
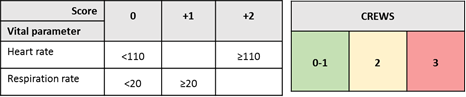


Table 3: New CREWS protocol for monitoring with Healhtdot on the ward

Indications for spot check monitoring

- Based on previous research, the Healthdot measurements can be relied upon, and no additional spot check monitoring will be routinely necessary for respiration and heart rate unless clinical doubt about the obtained value.
- Additional spot check monitoring for blood pressure, oxygen saturation and temperature may be requested by any member of the treatment team

Premature termination of the study

- In the event that the International Workload Scale (IWS) (13) is in the highest category at moment 4-5 for more than 50% of the staff.

## Withdrawal of individual subjects

Subjects of the core group can leave the study at any time for any reason if they wish to do so without any consequences.

## Replacement of individual subjects after withdrawal

Participants who withdraw their participation from the core group won’t be replaced.

## Follow-up of subjects withdrawn from treatment

N/A

## Premature termination of the study

In case the International Workload Scale (IWS) will be in the highest category of this scale for more than 50% of the staff at month 4-5 during phase two of this project.

# STATISTICAL ANALYSIS

# The statistical analysis at professional (nurses/ physicians) level will be performed by means of descriptive statistics. Each continuous parameter is analyzed for normality by the Shapiro-Wilk test and visually by a figure. Normality-based reporting will be done using medians and interquartile ranges (IQR) or means and standard deviations (SD). Frequencies and percentages are reported for categorical data. Each continuous parameter is checked for normality by the Shapiro-Wilk test and visually by a figure. Normality-based reporting is performed using medians and interquartile ranges (IQR) or means and standard deviations (SD).

## All analysis are performed using IBM SPSS Statistics 26.0 for Windows (IBM Armork, New York, USA) with 95% confidence interval and p < .05 as statistically significant.

## The core group consisting of nurses, physicians and investigators will conduct a structured analysis into the fidelity, feasibility and acceptance of the implementation and this information is processed and objectified by means of a Braun and Clarke thematic analysis.


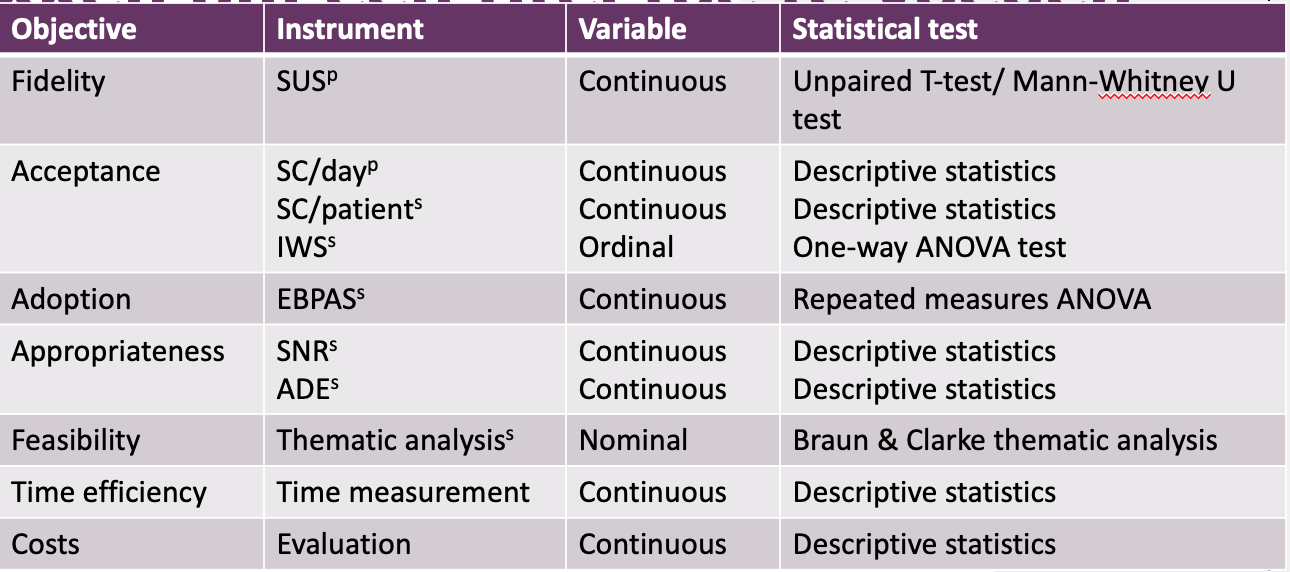


Table 4: psychometric properties and statistical analysis

## Other study parameters

N/A

# ADMINISTRATIVE ASPECTS, MONITORING AND PUBLICATION

## Handling and storage of data and documents

Paper research documents are stored in a folder in a lockable cabinet at the primary investigators office.

Digital data will be collected with use of research manager. Access to this system will be logged and encrypted.

## Monitoring and Quality Assurance

Monthly evaluation, starting after 3 months, with the focus group to assure the safety and quality of care during this project. Thereby, repetitive measurements of the IWS will provide important information regarding the safety of care and base of support from all involved.

## Amendments

Amendments are changes made to the research after a favourable opinion by the accredited METC has been given. All amendments will be notified to the METC that gave a favourable opinion.

A ‘substantial amendment’ is defined as an amendment to the terms of the METC application, or to the protocol or any other supporting documentation, that is likely to affect to a significant degree:

- the safety or physical or mental integrity of the subjects of the trial;
- the scientific value of the trial;
- the conduct or management of the trial; or
- the quality or safety of any intervention used in the trial.

All substantial amendments will be notified to the METC and to the competent authority.

Non-substantial amendments will not be notified to the accredited METC and the competent authority, but will be recorded and filed by the sponsor.

## Annual progress report

N/A for nWMO project

## Annual safety report

N/A for nWMO project

## Temporary halt and (prematurely) end of study report

N/A for nWMO project

## Public disclosure and publication policy

The results will be processed into one or more scientific articles that will be submitted to scientific journals in the field of monitoring, surgery, medical technology and general health care. Publication rights belong to the investigator. Research results are the property of the sponsor.

# REFERENCES

1. Joshi M, Ashrafian H, Aufegger L, Khan S, Arora S, Cooke G, et al. Wearable sensors to improve detection of patient deterioration. Expert Rev Med Devices. 2019;16(2):145-54.

2. Petersen JA, Antonsen K, Rasmussen LS. Frequency of early warning score assessment and clinical deterioration in hospitalized patients: A randomized trial. Resuscitation. 2016;101:91-6.

3. Gerry S, Bonnici T, Birks J, Kirtley S, Virdee PS, Watkinson PJ, et al. Early warning scores for detecting deterioration in adult hospital patients: systematic review and critical appraisal of methodology. BMJ. 2020;369:m1501.

4. Mok WQ, Wang W, Liaw SY. Vital signs monitoring to detect patient deterioration: An integrative literature review. Int J Nurs Pract. 2015;21 Suppl 2:91-8.

5. Majumder S, Mondal T, Deen MJ. Wearable Sensors for Remote Health Monitoring. Sensors (Basel). 2017;17(1).

6. Skivington K, Matthews L, Simpson SA, Craig P, Baird J, Blazeby JM, et al. A new framework for developing and evaluating complex interventions: update of Medical Research Council guidance. BMJ. 2021;374:n2061.

7. K. Mettert CL, C. Dorsey, H. Halko, B. Weiner. Measuring implementation outcomes: An updated systematic review of measures’ psychometric properties. Implementation Research and Practise. 2020.

8. S. Peres TP, R. Phillips. Validation of the System Usability Scale (SUS). SAGE Journals. 2013.

9. Aarons GA. Mental health provider attitudes toward adoption of evidence-based practice: the Evidence-Based Practice Attitude Scale (EBPAS). Ment Health Serv Res. 2004;6(2):61-74.

10. van Sonsbeek MA, Hutschemaekers GJ, Veerman JW, Kleinjan M, Aarons GA, Tiemens BG. Psychometric properties of the Dutch version of the Evidence-Based Practice Attitude Scale (EBPAS). Health Res Policy Syst. 2015;13:69.

11. Braun V, Clarke V. What can "thematic analysis" offer health and wellbeing researchers? Int J Qual Stud Health Well-being. 2014;9:26152.

12. Pickup L, Wilson JR, Norris BJ, Mitchell L, Morrisroe G. The Integrated Workload Scale (IWS): a new self-report tool to assess railway signaller workload. Appl Ergon. 2005;36(6):681-93.

13. R. Kramer AJ, M. Zelstra. How Busy is Too Busy? Validation of the Dutch Integrated Workload Scale. Proceedings of Fifth International Rail Human Factors Conference. 2015.

14. Patel V, Orchanian-Cheff A, Wu R. Evaluating the Validity and Utility of Wearable Technology for Continuously Monitoring Patients in a Hospital Setting: Systematic Review. JMIR Mhealth Uhealth. 2021;9(8):e17411.

15. Jacobs F, Scheerhoorn J, Mestrom E, van der Stam J, Bouwman RA, Nienhuijs S. Reliability of heart rate and respiration rate measurements with a wireless accelerometer in postbariatric recovery. PLoS One. 2021;16(4):e0247903.

16. Scheerhoorn J, van Ede L, Luyer MDP, Buise MP, Bouwman RA, Nienhuijs SW. Postbariatric EArly discharge Controlled by Healthdot (PEACH) trial: study protocol for a preference-based randomized trial. Trials. 2022;23(1):67.

17. van der Stam JA, Mestrom EHJ, Scheerhoorn J, Jacobs F, de Hingh I, van Riel NAW, et al. Accuracy of vital parameters measured by a wearable patch following major abdominal cancer surgery. Eur J Surg Oncol. 2021.

18. Downey CL, Brown JM, Jayne DG, Randell R. Patient attitudes towards remote continuous vital signs monitoring on general surgery wards: An interview study. Int J Med Inform. 2018;114:52-6.

19. Manetti S, Vainieri M, Guidotti E, Zuccarino S, Ferre F, Morelli MS, et al. Research protocol for the validation of a new portable technology for real-time continuous monitoring of Early Warning Score (EWS) in hospital practice and for an early-stage multistakeholder assessment. BMJ Open. 2020;10(12):e040738.

20. Chen L, Zheng H, Chen L, Wu S, Wang S. National Early Warning Score in Predicting Severe Adverse Outcomes of Emergency Medicine Patients: A Retrospective Cohort Study. J Multidiscip Healthc. 2021;14:2067-78.

21. G. Smith DP, P. Meredith, P. Schmidt, P. Featherstone. The ability of the National Early Warning Score (NEWS) to discriminate patients at risk of early cardiac arrest, unanticipated intensive care unit admission, and death. Resuscitation. 2013.

22. Duus CL, Aasvang EK, Olsen RM, Sorensen HBD, Jorgensen LN, Achiam MP, et al. Continuous vital sign monitoring after major abdominal surgery-Quantification of micro events. Acta Anaesthesiol Scand. 2018;62(9):1200-8.

23. Bellomo R, Ackerman M, Bailey M, Beale R, Clancy G, Danesh V, et al. A controlled trial of electronic automated advisory vital signs monitoring in general hospital wards. Crit Care Med. 2012;40(8):2349-61.

24. Breteler MJM, KleinJan EJ, Dohmen DAJ, Leenen LPH, van Hillegersberg R, Ruurda JP, et al. Vital Signs Monitoring with Wearable Sensors in High-risk Surgical Patients: A Clinical Validation Study. Anesthesiology. 2020;132(3):424-39.

25. M. Weenk MK, T. van de Belt, L. Engelen, H. van Goor, S. Bredie. Wireless and continuous monitoring of vital signs in

patients at the general ward. Resuscitation. 2019.

26. PLANNED. Translation and validation of the Dutch System Usability Scale. 2022.

# Attachments

SUS


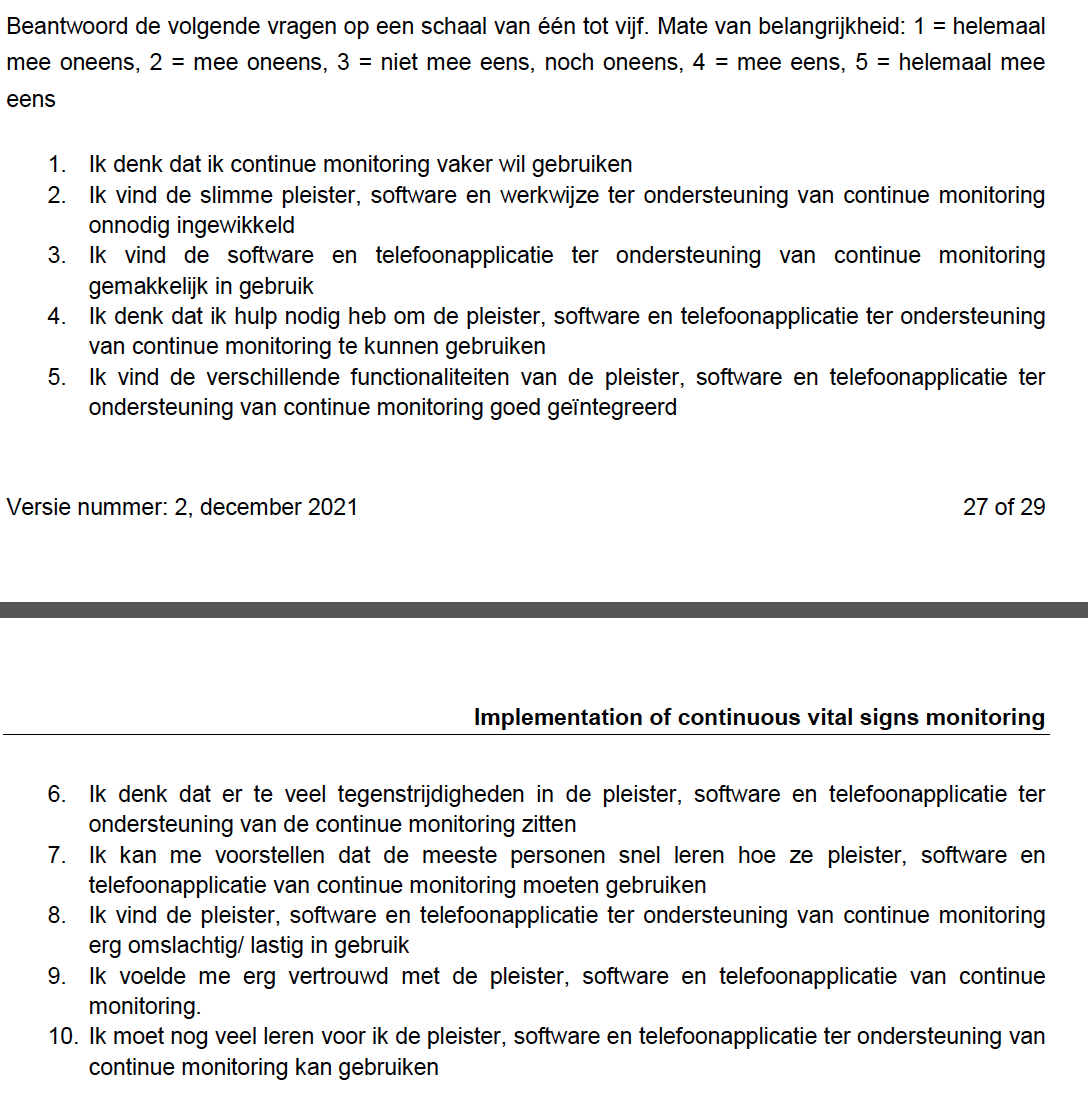


EBPAS


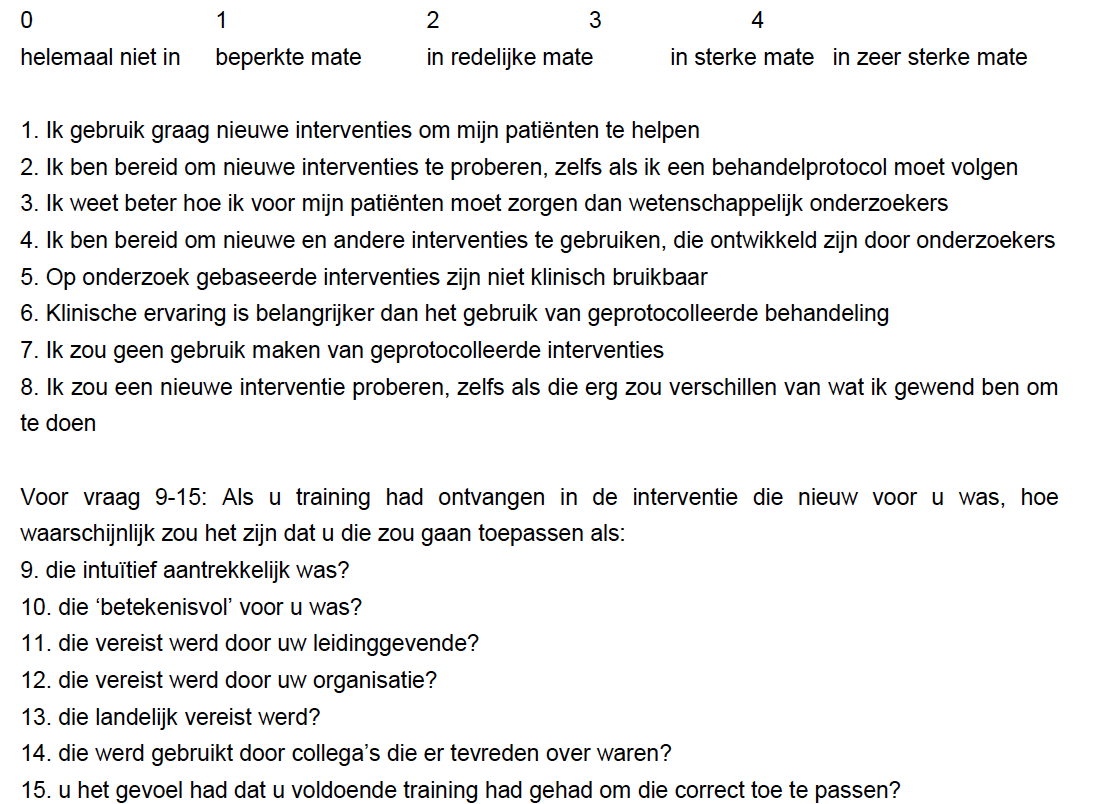


IWS (Dutch question options)


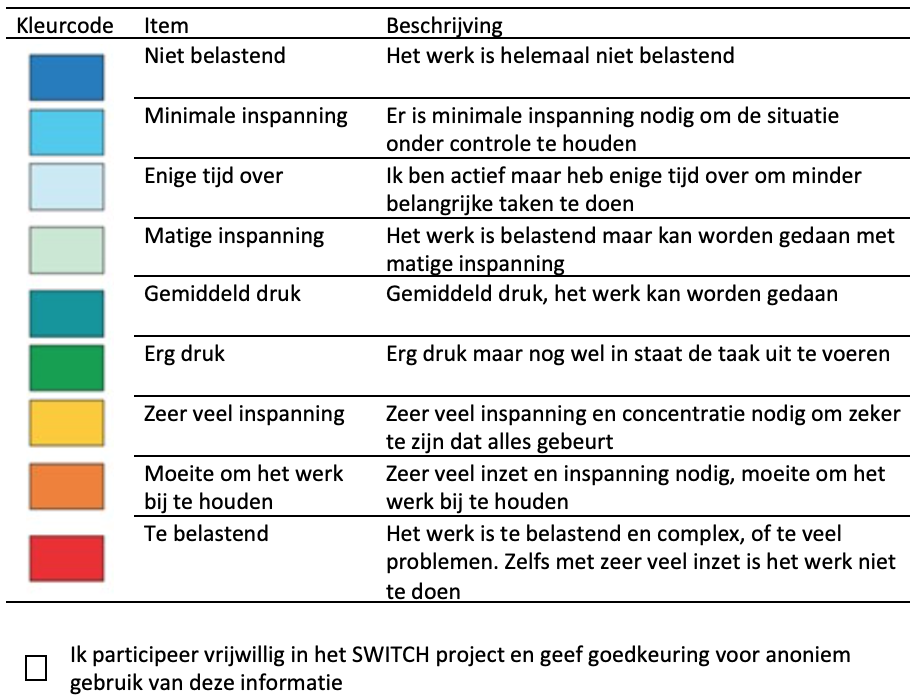

Supplement: S1 File — (DOCX) [file pone.0322472.s001.docx]
