# Supplementary material for: Implementation of a surgical ward innovation: Telemonitoring controlled by healthdot [SWITCH-trial PROTOCOL]
Source: PLoS One. 2025 May 7;20(5):e0322472. doi: 10.1371/journal.pone.0322472 (PMC12057926; doi:10.1371/journal.pone.0322472)
Supplement: Attachment — Submitted filename: Response to reviewers.docx [file pone.0322472.s003.docx]

Implementation of a Surgical Ward Innovation:

Telemonitoring Controlled by Healthdot (SWITCH-trial PROTOCOL)

**Response letter to reviewers’ questions**

Thank you for reviewing this manuscript and providing feedback. The questions below have been addressed and revised in the manuscript. The answers are highlighted in yellow.

Reviewer #1:

Is there any justification of the sample sizes of the nurses to complete IWS and the focus group?

- No sample size calculation was performed for the focus group; however, the number of participants was determined based on expert opinion and numbers comparable to other studies with focus group evaluations. For example, Enlöf et al.'s study, "Smart Glasses for Anesthesia Care: Initial Focus Group Interviews with Specialized Health Care Professionals" (February 2021), was conducted with 12 nurses and 4 physicians.https://www.sciencedirect.com/science/article/pii/S108994722030188X

If the analysis is descriptive, it is not meaningful to perform normality test and p values.

- Agree

The sentence of normality test is reiterated twice within the paragraph.

- Agree

Is there any analysis plan for the repeated measures of IWS?

- The IWS scores are measured daily and collected monthly. From these IWS scores, the average scores per month are calculated. These average scores will then be compared for statistical significance using an ANOVA-test for multiple means in SPSS.

Reviewer #2: I would like to commend the authors with their study and their clearly written trial-protocol.

- Thank you

There are two items that could benefit from clarification, in my opinion, which are:

Did the nursing staff had prior experience with continuous remote monitoring? Were they given a training prior to the trial?

- The nursing team on this surgical oncology ward has experience with smart monitoring and this specific device, as it was validated on the same ward in an earlier clinical trial. However, there is a gap of at least three years between these studies, leading to significant changes in the composition of the nursing team. Consequently, a large portion of the team, particularly the new and younger nurses, were using the device for the first time. The experienced nurses took on the role of mentoring their colleagues, which contributed to the successful implementation.

Prior to the study and during the initial (pre-implementation) phase, all nurses completed an e-learning course and participated in several hands-on training sessions to prepare for the implementation as stated in methods.

Can you clarify the timeframe of the primary outcome? It now states that the IWS is measured throughout the study, but perhaps it would be wise to specifically state if that's once a month, twice-weekly, etc.

- The IWS was completed daily by the nurses working on the ward during their shifts. Therefore, it was a daily measurement that was collected and evaluated monthly.

Reviewer #3: Dear authors,

thanks for the opportunity to review the interesting paper. As a nurse, I am experienced in postoperative nursing care and I am experienced in continuous vital signs monitoring using a wearable device which captures 6 vital signs (RR, SPO2, HR, PR, blood pressure and skin temperature). This background provides me with an opinion regarding implementing, using and researching continuous monitoring. I know that a lot of work still needs to done, to move forward in inhospital continuous monitoring of vital signs. That gives me the following critical notes to the study that the authors propose in this paper.

First, I wonder why the authors choose an device that does not provide all relevant vital parameters that are suggested by the society of critical care medicine (2023) when nurses/physicians try to recognize clinical decline of patients. I would like to ask the authors to elaborate on this in the background. Furthermore, the study does not ‘assess wearables’, but evaluate one device, so please be explicit on this in the aim and abstract.

- The Early Warning Score (EWS) has been in use for approximately 30 years since its introduction and is based on multiple vital parameters, as you describe. Over time, the EWS has been revised, but only to a limited extent. In a previous study, van der Stam JA, Mestrom EHJ, Scheerhoorn J, Jacobs F, de Hingh I, van Riel NAW, et al. Accuracy of vital parameters measured by a wearable patch following major abdominal cancer surgery. Eur J Surg Oncol. 2021, conducted at our center on the same ward, the accuracy and clinical reliability of EWS measurements were compared with those of the Philips Healthdot. This study found that, despite a reduction in the number of parameters, respiratory rate and heart rate were reliable predictors of clinical deterioration. The data analysis demonstrated that the Healthdot's predictive capability was at least as good as that of the EWS.

Based on these findings, the CREWS protocol was developed and used during the SWITCH study. These findings are supported by literature, which identifies respiratory rate and heart rate as the best predictors of clinical decline. Given the experience with and validation of this device, it was chosen for implementation in this study. However, the market is evolving, and new devices with capabilities for additional parameters have become available, offering further options for application.

Second, capturing vital signs is required for evaluation of clinical condition. That in itself requires effort and time, obviously; ensuring the safety of patients is fundamental to nursing care. Measuring vital signs is the basis for clinical reasoning, to interpret the condition, and set targets for treatment and evaluations with physicians/ICU team. Introducing this technology will change the way this work is done. The authors choose to evaluate the reduction workload for nurses at outcome of this change but why? Maybe it reduces workload of ICU’s by reduced IC admission, but increases workload on general wards due to earlier recognition and subsequent initiation of treatment? Do you take this time also into account? As well as using CREWS besides the regular EWS for interpretation? Is that not a point of interest: the work process instead of workload? Therefore, I would suggest to the authors to elaborate more in the introduction on the role of nurses in either vital signs measurement as well as on how the use of wearables for vital signs measurement is already integrated or used in nursing practice. That may clarify to specific need for this research question.

- The primary outcome is workload reduction, given the implementation of a new device and a different way of working that has already been validated and proven reliable. Considering current challenges on the hospital wards, such as staff shortages, staff burn-out, and increasingly complex patients with comorbidities, the implementation is focused on improving efficiency and reducing workload. The initial phase involves the use of both monitoring methods, which temporarily adds to the daily tasks of the nursing staff. However, this is only for a period leading up to the transition to monitoring solely with the Healthdot. During this phase, IWS measurements, manual time measurements of monitoring rounds, and workload experiences are evaluated during focus group sessions to monitor and assess the workload during the different phases of implementation. Time is a crucial metric and is measured both qualitatively and quantitatively through focus group analyses, IWS questionnaires, and manual measurements of the duration of monitoring rounds. The elimination of standard EWS measurements during the second phase is precisely calculated, allowing for the determination of time savings, as the average EWS duration is also measured. Alarm fatigue analysis, along with focus group analyses, will illustrate whether there is excessive alarming or additional workload due to the availability of continuous monitoring data.

A summary of the above is added to the introduction.

Thirdly, I do have some suggestions in more detail.

Abstract

~~-~~background: ‘require a significant amount’: what is required?

- Agree, Adjusted

-wearable devices are effective on what? do you mean these are adequately able to capture vital parameters in an accurate manner?

- Is added in introduction

-workload: what does workload mean, and what provokes the workload? the declining patient or manually capturing vital signs?

- ‘ The workload for nursing staff consists of many more factors than assessing EWS only and is a difficult to retrieve multidimensional variable.’ is added.

-unclear in the abstract what the characteristics of the device are, please explain

- Is explained in Methods section.

-implementation strategies are missing? please elaborate briefly

- Quite demanding to fit into an abstract, it is already described further on.

Background:

-authors stresses the need to reduce workload/efforts of nurses, and argue that monitoring is an option for earlier discharge by transmurally monitoring. Who is the professional that perform the surveillance of continuous monitoring either clinically or transmurally?

- In this study focus is on hospitalized patients only.

-clear state of science is lacking, what does systematic reviews says on devices, as well as what does studies says on effectiveness of monitoring on earlier recognition of decline? what does studies report on implementation factors, is their any knowledge on successfully adopted and well-embedded continuous monitoring?

- This is corrected

Aim:

-plural form is used for wearables, however, only one device is evaluated

- This is corrected

Methods

- theory on implementation and subsequent implementation strategies are missing.

- Proctor based theory and discussion with the nursing staff guided towards the subsequent implementation as explained in this report.

Finally, I wonder why the authors choose to report results separately, I think that results and discussion will increase the value of the study as it is involves only one wearable in one setting.

- For the sake of clarity, first description of ‘ results’ followed by ‘ discussion’ approach was used.
