# Supplementary material for: Implementation of a surgical ward innovation: Telemonitoring controlled by healthdot [SWITCH-trial PROTOCOL]
Source: PLoS One. 2025 May 7;20(5):e0322472. doi: 10.1371/journal.pone.0322472 (PMC12057926; doi:10.1371/journal.pone.0322472)
Supplement: Attachment — Submitted filename: Response to comments to Author 4.docx [file pone.0322472.s005.docx]

**Response to comments to Author**

6. Review Comments to the Author

Please use the space provided to explain your answers to the questions above and, if applicable, provide comments about issues authors must address before this protocol can be accepted for publication. You may also include additional comments for the author, including concerns about research or publication ethics.

You may also provide optional suggestions and comments to authors that they might find helpful in planning their study.


(Please upload your review as an attachment if it exceeds 20,000 characters)

Reviewer #1: If the analysis is descriptive, it is not meaningful to perform normality test and p values.
- Agree
The sentence of normality test is reiterated twice within the paragraph.
- Agree

These two issues were not edited in the statistical analysis section.

Response:

By mistake these issues were not edited in the statistical section. I have changed these issues and hope this will now be resolved accordingly.
